# Molecular Contrast Optical Coherence Tomography and Its Applications in Medicine

**DOI:** 10.3390/ijms23063038

**Published:** 2022-03-11

**Authors:** Ancong Wang, Wenliu Qi, Tianxin Gao, Xiaoying Tang

**Affiliations:** School of Life Science, Beijing Institute of Technology, Beijing 100081, China; awang@bit.edu.cn (A.W.); 3220201324@bit.edu.cn (W.Q.); gtx@bit.edu.cn (T.G.)

**Keywords:** molecular imaging, optical coherence tomography, molecular contrast agent, molecular contrast OCT, nanoparticles

## Abstract

The growing need to understand the molecular mechanisms of diseases has prompted the revolution in molecular imaging techniques along with nanomedicine development. Conventional optical coherence tomography (OCT) is a low-cost in vivo imaging modality that provides unique high spatial and temporal resolution anatomic images but little molecular information. However, given the widespread adoption of OCT in research and clinical practice, its robust molecular imaging extensions are strongly desired to combine with anatomical images. A range of relevant approaches has been reported already. In this article, we review the recent advances of molecular contrast OCT imaging techniques, the corresponding contrast agents, especially the nanoparticle-based ones, and their applications. We also summarize the properties, design criteria, merit, and demerit of those contrast agents. In the end, the prospects and challenges for further research and development in this field are outlined.

## 1. Introduction

Molecular imaging is defined as a real-time, noninvasive way to visualize, characterize and even quantify the biochemical events that occur at the cellular and molecular level within living organisms, including patients. Therefore, it can depict the internal physiological and pathological processes, such as molecular actions and pathways, to reveal the microenvironmental abnormalities in the living subjects before gross pathological and anatomical changes come up. In general, researchers utilize particular molecular imaging instrumentations, algorithms, and mathematic models to study the sample characteristics alone or with imaging agents. Although in its infancy, molecular imaging has become a vital tool to study complex biochemical phenomena building up to form the basis of disease, which facilitates early detection, monitoring, and optimized treatment of diseases such as cancer and cardiovascular disease. Scientific researchers also use molecular imaging to improve drug discovery and validation.

There exists a wide range of classical molecular imaging modalities, including positron emission tomography (PET), single-photon emission computed tomography (SPECT), computed tomography (CT), magnetic resonance imaging (MRI), ultrasound (US), and a series of optical imaging methods [1,2,3,4,5,6]. Every modality has its key strengths and limitations in spatial and temporal resolution, penetration, sensitivity, cost, and so on. A detailed comparison of these modalities is given in Table 1.

Optical imaging contains a series of techniques such as fluorescence imaging, bioluminescence imaging, photoacoustic imaging, Raman imaging, and optical tomography. Sensitivity and multiplexing capabilities are major strengths of these techniques, but they are relatively expensive. Besides, these modalities have either high spatial resolutions or good penetration depths. Optical coherence tomography (OCT) is a noninvasive optical imaging modality that provides real-time 2D/3D scans depicting morphological structures of living tissue with unique micron-scale resolution and 2~3 mm depth penetration [7]. It just fills the niche between confocal reflectance microscopy and diffuse optical tomography. Compared to other optical imaging methods, OCT can generate sharp, reliable, and cost-effective images using light fluences far below the recommendation of the American National Standard (ANSI). A detailed description of OCT concepts and characteristics will be given in the next section. Due to its advantages, OCT has been commercially available for a wide range of research and clinical applications in ophthalmology, cardiology, dermatology, odontology, gastroenterology, and oncology [8].

OCT is usually considered more of a structural imaging method than a functional imaging method, but its popularity raises a strong demand for molecular imaging with OCT. Since the early 1990s, reports on nanomedical research using OCT have been gradually increasing. Conventional OCT has a fairly restricted capacity to track biochemical distribution and changes within living organisms. It is because OCT cannot distinguish the signals from different molecules and the background nor detect incoherent processes like Raman scattering or fluorescence emission. To address this, significant effort has been taken to develop sophisticated OCT strategies that can provide additional ex vivo, in vitro, and in vivo molecular information [9].

To image living subjects with molecular selectivity commonly requires the use of specific labels. In most cases, molecular contrast OCT techniques are combined with contrast agents to enhance their diagnostic capabilities. The contrast agents can be endogenous or exogenous. Among these, nanoparticles are peculiarly suitable for reflection-based or scattering-based imaging methods. Benefitted from the development in nanomaterials, many engineered nanoparticles have been reported as nonspecific or targeted contrast agents for OCT.

In this paper, we reviewed the studies of molecular contrast OCT techniques and their applications. Given the limited space available, we had to restrict the content to the main principles, concepts, and some selected applications in the biomedical field. To be specific, we firstly describe the concepts and characteristics of general OCT systems. Next, we present the current progress of all molecular contrast OCT extensions, corresponding contrast agents, and applications, primarily focusing on the work dedicated to combining OCT with nanoparticle contrast agents in the field of nanomedicine. Finally, we briefly overview the advantages and disadvantages of those molecular OCT imaging methods, contrast agents and explore the development trends in the future.

## 2. Conventional OCT

All molecular contrast OCT techniques are derived from conventional OCT. Analogous to US imaging, conventional OCT provides cross-sectional images of a sample, except that it uses near-infrared light instead of ultrasound. Due to the magnitude of the speed of light, optical echo pulses can only be captured indirectly using low-coherence interferometry.

### 2.1. Background

**Figure 1:**
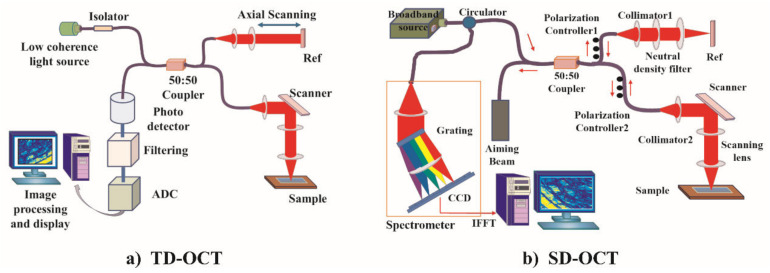
Schematic diagram of generalized OCT systems. A TD-OCT and a spectral-domain OCT are demonstrated in (**a**,**b**), respectively. The arrow in (**a**) indicates the movement of the reference arm. The arrows in (**b**) show the light travel paths. Adapted with permission from [11]. Copyright 2018 J-STAGE.

The first developed system is time-domain OCT (TD-OCT) which contains a low time-coherence, broad bandwidth light source. As Figure 1a shows, the emitted light is split into two same beams through a Michelson interferometer that is usually composed of optical fiber coupler. The beam transmitted to the sample is called the probing beam or scanner, while the other beam is the reference beam which travels to a mirror on the reference arm. The backscattered light from the sample and the reflected light from the reference arm meet at the interferometer and can produce a stable interference pattern if they have traveled the same distance. This pattern is recognized as the intensity of the sample on the probing path at the given depth. By moving the reference mirror, TD-OCT changes the light path for interference and gives rise to the intensity profile of the sample, namely A-scan. Cross-sectional images (B-scans) are achieved by combining continuous A-scans. Its scan speed is restricted by the moving speed of the reference arm.

There have been OCT techniques that increase the imaging speed significantly, which are known as frequency-domain OCT (FD-OCT). With a similar scheme, FD-OCT collects all the scattering information of a whole A-scan at the same time without moving the reference arm. There are two types of FD-OCT: spectral-domain OCT (SD-OCT) and swept-source OCT (SS-OCT). The former needs a broad bandwidth light source for imaging. It analyzes the spectrum of backscattered light with a spectrometer and transforms the results into information related to the depth of the structure in the tissue using the Fourier principle. The schematic diagram of a generalized SD-OCT system is given in Figure 1b. The latter encodes the optical frequency in time with a frequency swept, narrow bandwidth laser source which rapidly sweeps the source wavelength over a broad wavelength range. Collected spectral data are inverse Fourier transformed to recover the spatial depth-dependent information. Hence, the line rate determines the image acquisition speed of FD-OCT, and the axial scan time could be less than 0.1 microseconds [10]. These techniques improve the imaging speed a hundredfold while raising the sensitivity.

The depth resolution of an OCT system is defined by the source coherence length, with typical values of 6–15 µm in air, and the lateral resolution by probe beam size diameter. Nano-sensitive OCT is a modern design that provides extra sensitivity to nanometer-scale structural changes or measurements [12,13,14,15,16,17]. Though depicting cellular or sub-cellular level structures remains a challenge, the imaging resolution of OCT is still at least 20 times finer than other in vivo 3D imaging techniques, such as ultrasound. The interferometry guarantees that OCT selectively measures only the backscattered light directly coming from the interested surfaces in tissue while rejecting most photons that scatter multiple times before arriving or come from any other incoherent light sources. It is the key to building clear, high-resolution 3D images of thick samples with minimum background noise, yet it also prevents OCT from straightforwardly taking advantage of molecular processes such as bioluminescence emission, fluorescence emission, and Raman scattering. Fundamentally, OCT images illustrate the intensity of backscattered probe light which is derived from variations of the refractive index of a sample. Unfortunately, most bio-molecular species cannot be simply distinguished from each other based on their refractive indexes only. This feature makes it a challenge to develop molecular contrast OCT systems. However, given the rapid penetration of OCT into research and clinical practice, robust molecular imaging extensions are still strongly desired in addition to high-resolution morphological imaging from OCT.

### 2.2. Engineered Particle-Based Method and Its Applications

It is a straightforward way to acquire molecular images of biological tissues by introducing appropriate contrast agents into the target region to change the intensity of backscattered light. This kind of contrast agent should have high reduced scattering coefficients or absorption coefficients. The conventional OCT images of the sample before and after applying contrast agents are subtracted to achieve the distribution of contrast agents. Lee et al., demonstrated a series of encapsulated microspheres for molecular contrast OCT imaging [18]. In this application, microspheres (0.2~15 µm in diameter) have been engineered with 50 nm thick protein shells, and their shells and cores can be mixed with different materials and nanoparticles. The microspheres with melanin, gold, or carbon nanoparticles were synthesized and all these microspheres provided a higher degree of light scattering than pure microspheres or biological tissues. In this study, mouse models were injected with gold nanoparticle mixed microspheres, and their livers were scanned with OCT. Compared with the control group, OCT images for mice treated with the contrast agent were strongly enhanced. Although these contrast agents are non-targeted, their protein shells can be functionalized to probe specified molecules.

From then on, nanoparticles such as gold nanorods, gold nanocages, and TiO_2_ nanoparticles have been reported for molecular contrast OCT [19,20,21,22,23,24,25,26,27,28,29,30,31,32,33,34,35,36]. For example, Chen et al., presented the first scattering-dominant gold nanoparticles, nanocages, for molecular contrast OCT imaging [37]. However, the potential to use contrast agents to selectively enhance particular cells or molecules in living tissues was not explored until fairly recently. Hu et al., demonstrated that conventional OCT can detect individual cells suspended in biological fluids with specifically designed gold nanoshells (GNSs) [38]. The GNSs had silica cores of 200 ± 10 nm and gold shells of about 15 nm, which exhibited a strong backscattering cross-section at 1300 nm. The performance of this method was validated using both adherent and nonadherent cells: Hela cells and Jurkat cells. These cells were incubated in a culture medium containing the same concentration of GNSs and then scanned using conventional OCT. The OCT images of the tubing filled with pure phosphate-buffered saline (PBS), PBS with GNSs, normal Hela and Jurkat cells, and the Hela and Jurkat cells incubated with GNSs are demonstrated. The spot intensity distribution indicated that this method can significantly enhance the backscattered light from the cells incorporated with GNSs. It is possible to obtain the population of the living cells that incorporated GNSs using a simple intensity analysis. The scanning results from dark-field microscopy and transmission electron microscopy were in agreement with this conclusion. Kim et al., demonstrated that surface plasmon-resonant gold nanoparticles are able to enhance the contrast of OCT images [39]. In this study, antibody-conjugated PEGylated gold nanoparticles of 71 nm in diameter were prepared and applied to the hamster’s cheek pouch for 10 min. The ultrasonic force was applied to facilitate nanoparticles dispersion into a sample. Results suggested that the OCT contrast level is increased by about 150%.

**Figure 2:**
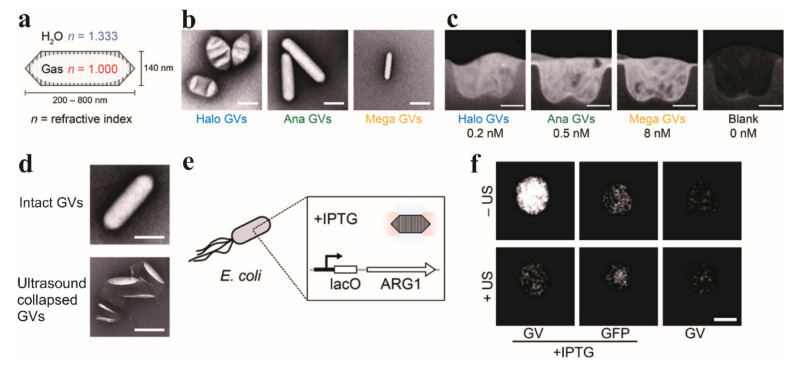
(**a**) Schematic drawing of a GV, the gas-filled interior of which has a refractive index (red) different from that of the surrounding H_2_O (blue). (**b**) Representative transmission electron microscopy (TEM) and (**c**) OCT images of GVs from *Halobacterium salinarum* NRC-1 (Halo), *Anabaena flos-aquae* (Ana), and *Bacillus megaterium* (Mega). GVs were embedded in agarose hydrogel for OCT imaging. (**d**) Representative TEM images of Ana GVs before and after ultrasound treatment. (**e**) Diagram of the IPTG-inducible expression of ARG1 GVs inside *E. coli*. (**f**) Representative C-scans of colonies expressing GVs or green fluorescent proteins (GFPs), in the presence or absence of the inducer, and subjected to ultrasound or left intact. Adapted with permission from [40]. Copyright 2020 American Chemical Society.

Previous work focused on synthetic particles which generate strong NIR scattering, but these particles have difficulty in entering living tissues and coupling to intracellular processes. Lu et al., introduced the first genetically encodable targeted molecular imaging agents for conventional OCT based on gas vesicles (GVs) [40]. These GVs are gas-filled nanostructures with 2 nm-thick protein shells, which were produced inside genetically programmed microbes. Three different types of GVs were demonstrated in this study, which were assembled by *Anabaena flos-aquae*, *Halobacterium salinarum* NRC-1, and *Bacillus megaterium* as Figure 2a–c show. They all produced significant NIR scattering at nanomolar concentrations. Their protein shells can be modified for various purposes through genetic engineering. Figure 2e,f demonstrate that GVs can act as reporter genes in living *Escherichia coli* that were engineered to express ARG1. Moreover, GVs will collapse and disappear under the pressure of ultrasound pulses which can facilitate the dynamic OCT contrast generation (Figure 2d).

The advantage of this method is that it requires no modification of the conventional OCT scheme and is easy to apply. There is also a wide range of particles that can provide molecular OCT contrast under certain conditions. Its disadvantage is also apparent because it is difficult to separate the scattering changes caused by exogenous contrast agents from the scattering variation from the sample itself. Such changes are not generally detectable using only reflectivity measurements unless the scattering contribution from the contrast agent is sufficiently large. There are only a few reports of targeted molecular OCT contrast agents so far, but many nanoparticles such as protein and gold nanoparticles can be functionalized as targeted probes in the future. Much effort has been put into developing techniques to acquire molecular imaging using OCT. As a result, various molecular contrast OCT schemes and applications have continued to be reported.

## 3. Pump–Probe OCT and Its Applications

**Figure 3:**
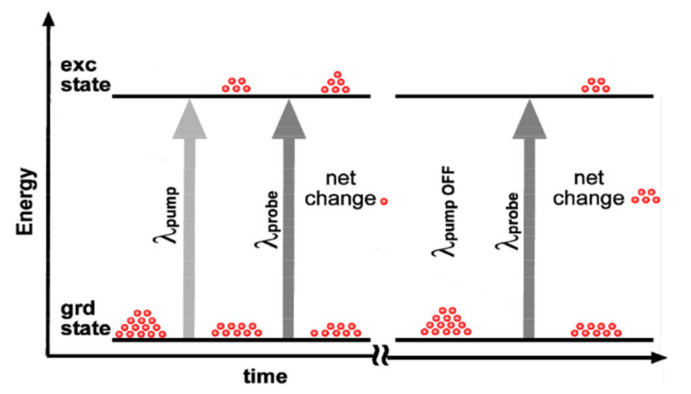
Energy level diagram for a ground-state recovery pump–probe experiment. The pump radiation transfers the ground state population into the excited state. The probe radiation then measures the population transfer induced by the pump, which is manifested as a reduction in ground state absorption and an increase in excited state stimulated emission. Adapted with permission from [43]. Copyright 2006 Optical Society of America.

Pump–probe OCT (PP-OCT) integrates pump–probe spectroscopy with OCT to measure the distribution and concentration of molecular contrast agents in a sample [41]. Pump–probe spectroscopy is a standard technique to measure ultrafast electronic dynamics using ultrashort laser pulses. This technique involves two beams: pump and probe. As the pump beam hits a sample, a range of physical phenomena can be induced to change the optical properties of the sample, and the probe beam is applied to monitor the change before and after the pump excitation as Figure 3 presents. PP-OCT schemes introduce an extra, modulated light source in the conventional OCT probe-arm as the pump beam and use the original OCT probe beam to measure the change in the sample. During the PP-OCT scan, a baseline OCT image is acquired when the pump light is off. Then, the second OCT image is acquired after the pump light is on, in which the intensities are expected to be changed. The differential intensities between these two images are proportional to the distribution of excited molecules in the sample. In the end, PP-OCT transfers the optical changes into molecular signals using a specially designed algorithm. There are various phenomena to produce pump–probe signals, such as stimulated emission, photodegradation, photoisomerization, and most commonly, transient absorption [42].

The first implemented time-domain PP-OCT system provides molecular information based on the transient absorption of methylene blue (MB) caused by pump excitation in a sample. The preliminary data demonstrated the location and population of MB in multilayer phantoms [41]. MB was chosen as the contrast agent because it has an absorption peak at about 660 nm region in the singlet state, while in the triplet–triplet state, it has an absorption band centered at 830 nm which is overlapped with the probe light of standard OCT. It means, with an excitation beam, methylene blue changes from transparent to strongly absorbent at 830 nm. The presence of MB in phantoms is clearly shown in the experiment, but this method has noticeable limits. MB has a very short transient absorption lifetime of about 2 µs, which provides only a tiny window for imaging. To obtain high-quality signals, it has to repeatedly scan the same position over 100 times, which not only significantly slows down the imaging speed, but also raises the incident laser fluence above the damage threshold [44].

To optimize the imaging window, Yaqoob et al., proposed a PP-OCT scheme using indocyanine green (ICG) as the imaging agent and demonstrated the molecular contrast in tissue phantoms and *Xenopus laevis* samples [45,46]. ICG in the ground state has an absorption spectrum that peaks at the NIR region (810 nm) when mixed with human plasma or albumin. With pump light excitation, ICG will be gradually photo-bleached into a longevous, transparent isomer. The lifetime of this alteration is over 10 ms which offers a much longer imaging window than MB. With the prior knowledge of ICG bleaching dynamics, the depth-resolved distribution of ICG in the sample can be determined. This scheme is able to study animal models for the visualization of blood vessels and body cavities. ICG improves the sensitivity of PP-OCT. Another advantage of ICG is that it is FDA-approved for medical diagnosis and therapy. However, this scheme still suffers from its inefficient pump–probe mechanism and cannot generate sufficient sensitivity for clinical applications. The imaging speed is so slow that it is not suitable for time-critical applications. For example, a PP-OCT scan with 10 μM ICG solution as contrast agents will take over 350 s in order to satisfy the laser illumination safety standard for long time skin exposure (300 mW/cm^2^).

Previous methods probe the sample after pump laser has driven the molecule to a steady triplet or isomeric state, but Applegate et al., presented an optimized PP-OCT technique that can measure the transient absorption changes of electronic transitions over an ultrashort period before a steady state is achieved [43]. It is sensitive to any molecular chromophore since it does not rely on efficient isomeric transitions or spin-forbidden. The new method can garner two different molecular properties: the transient absorption spectrum and the ground state recovery time. The transient absorption magnitude of a chromophore can be mapped with its absorption spectrum using mathematic models. As the probe intensity attenuation is proportional to the ground state molecule population during the pumping process, the ground state recovery time can be calculated easily. Moreover, the ground state recovery calculation requires prior knowledge of only one excited state instead of all states. This feature enables PP-OCT to detect multiple contrast agents simultaneously. Experiment results prove that the new method can separate the signals from rhodamine 6G and hemoglobin in a mixed solution. Despite all the advantages, the laser fluence of this method is still above the ANSI-suggested limitation.

**Figure 4:**
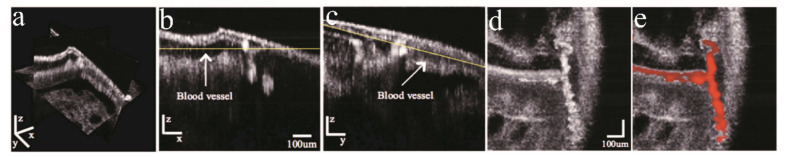
(**a**) Volumetric OCT image of tadpole. (**b**) Cross-sectional image in the x, z plane. (**c**) Cross-sectional image in the y, z plane. (**d**,**e**) are cross-sectional cuts along the yellow lines depicted in (**b**,**c**). (**d**) OCT image and (**e**) OCT image overlaid with the pump–probe signal from blood. Adapted with permission from [49]. Copyright 2015 WILEY-VCH Verlag GmbH & Co. KGaA, Weinheim.

The concern of the light fluence and the sensitivity was not relieved until the FD-OCT scheme is used in PP-OCT approaches. Jacob et al., presented a two-color Fourier-domain PP-OCT system which is at least 1000 times faster than previous ones [42]. In this study, melanin was used as the contrast agent for the ex vivo molecular imaging of a human hair in chicken meat and a porcine iris. Experimental data demonstrated that it has a comparable penetration depth as conventional OCT. The pump light fluence was further reduced to meet the ANSI limitation by raising the A-line ratio at a cost of SNR loss, but the image quality is still acceptable for animal model studies. Afterward, Carrasco-Zevallos et al., demonstrated that this PP-OCT approach can acquire ex vivo images of Zenopus tadpole vasculature using MB and hemoglobin as molecular contrast agents separately and mixed [47,48]. In the end, they get the first in vivo PP-OCT image which depicts the vasculature system in living *Xenopus laevis* [49]. Hemoglobin in the blood is a natural contrast agent. In this study, the performance of PP-OCT was compared with Doppler OCT and phase-sensitive OCT and proved to be the most suitable for the twisted vessel description. In Figure 4, the OCT and PP-OCT images of a tadpole are depicted. The PP-OCT signals clearly exhibited the vessels in the tadpole with little noise.

A lot of studies have been done to explore novel efficient contrast agents for PP-OCT imaging. Its performance should be further verified. In 2016, Kim et al., synthesized 2.5 μm diameter hollow poly lactic-co-glycolic acid (PLGA) microspheres containing 0.01% (*w*/*v*) aqueous MB as a contrast agent for PP-OCT [50]. It can enhance the PP-OCT signals by increasing the scattering surfaces and slowing down the MB bleaching. Microencapsulation lowers the toxicity of MB by preventing 92.8% of methylene blue from turning into colorless leucomethylene blue during the imaging and reducing 78.3% of the singlet oxygen generation from the excited MB. Another benefit of the PLGA shell is that it can easily be functionalized with ligands to target molecular biomarkers while keeping the excitation dynamics of contrast agents for PP-OCT imaging. Years later, they decreased the size of MB-filled PLGA particles into nanoscale (~84 nm) which can penetrate into tissues deeper than before [51,52,53]. The particle surface was also functionalized to target biomarkers in atherosclerotic plaque including vascular cell adhesion molecules and apoptotic macrophages. Validation results confirmed the effectiveness of the new imaging agent.

PP-OCT is a promising molecular imaging technique to locate molecules in complex environments such as biological tissues. A desirable feature of PP-OCT is that its contribution to light attenuation variation is highly distinguishable which requires no compromise between the spatial resolution and the spectral resolution. Another major advantage is the multiplexing capability of PP-OCT which can detect several mixed molecular species in the sample simultaneously. The disadvantages include its complicated and expensive optical setups and the concern about the safety profile. With low pump pulse energy, a PP-OCT scan has to balance between signal quality and imaging speed. Theoretically, any molecule capable of absorbing light is a potential contrast agent of PP-OCT. Reported endogenous contrast agents include melanin and hemoglobin. There are also many exogenous molecules, such as MB and ICG, which are compatible with PP-OCT imaging. The PLGA-based microencapsulation technology can encapsulate these dyes into nanoparticle-based contrast agents which are versatile, robust, and have better chemical and photo-stability. Up to now, the primary challenge is that the dyes used for pump–probe OCT imaging should have a relatively long-lived triplet state so that the demanded pump light energy will be lower and the imaging time window could be longer. In the future, the PP-OCT technique may benefit the in vivo vasculature imaging and the blood oxygen saturation measuring of humans. Its potential clinical applications include atherosclerosis, tumors, and choroidal neovascularization studies.

## 4. Pump Suppression OCT and Its Applications

**Figure 5:**
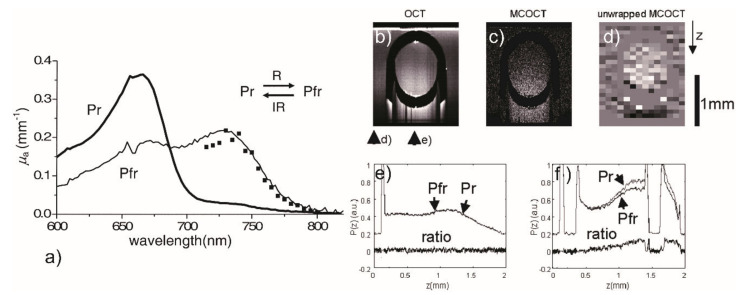
(**a**) Absorption spectra of the two states of PhyA. (**b**) The 750 nm OCT B-scan with PhyA in Pr state (1.5 mm wide × 2 mm deep); the OCT B-scan with PhyA in Pfr state appears very similar (not shown). (**c**) molecular contrast OCT differential scan. (**d**) Unwrapped MCOCT scan. (**e**) A-scans with PhyA in Pr and Pfr state extracted from the locations indicated by the arrows in b. (**f**) A-scans with PhyA in Pr and Pfr state extracted from the locations indicated in b. Adapted with permission from [9]. Copyright 2005 American Society for Photobiology.

Pump suppression OCT has a similar design as PP-OCT but utilizes different molecular transition mechanics. Unlike the latter, the new optical switch suppression technique detects the conformational change of molecules before and after the pump suppression. There are only two types of pump suppression OCT contrast agents reported so far, which are bacteriorhodopsin (bR) [54] and phytochrome A [55]. Such kinds of molecules can reversibly change from one stable state to another through pump excitations. The absorption wavelength regions of a molecule in these two states are distant, and one of the regions overlaps with the OCT probe light spectrum. Therefore, an OCT probe can scan at different stages to calculate the 3D distribution of molecular contrast agents. Yang et al., presented a pump suppression OCT system and demonstrated clear signals from bR solution samples during the scan [54]. In the original state, bR has an absorption peak in the javascript:void(0)red-green region of the spectrum. When exposed to 500–800 nm pump light, bR transits into a new state whose absorption peak locates in the ultraviolet region of the spectrum. The new state can exist for tens of milliseconds. One year later, they reported the use of phyA as a contrast agent for molecular contrast imaging [55]. The absorption spectra of the two states of phyA have significant differences, as Figure 5a demonstrates, and thus can be distinguished. The OCT image, pump suppression OCT, and unwrapped images of a phyA solution-filled tube are given in Figure 5b–d. The comparison of an A-scan passing through the tube and another A-scan that does not pass the phyA solution is given as well.

The advantage of pump suppression OCT is that it obtains an imaging window at least six orders of magnitude longer than PP-OCT. As a bonus, the pump light used in this scheme has a significantly low intensity. Its contrast agents also bring in numerous advantages. First and foremost, all bacteria species and even mammalian cells could express proteins like bR and may be detected through pump suppression OCT imaging. Second, these contrast agents are stable in both states. However, the range of possible imaging agent candidates is still restricted. Currently, there are no nanostructured contrast agents yet. Both existing contrast agents are proteins, so research into the development of protein nanoparticles or protein combined nanoparticles as contrast agents for pump suppression OCT is necessary [56,57]. More candidates may come forth with the rapidly growing optically switchable material research.

## 5. Nonlinear Interferometric Vibrational Imaging and Its Applications

**Figure 6:**
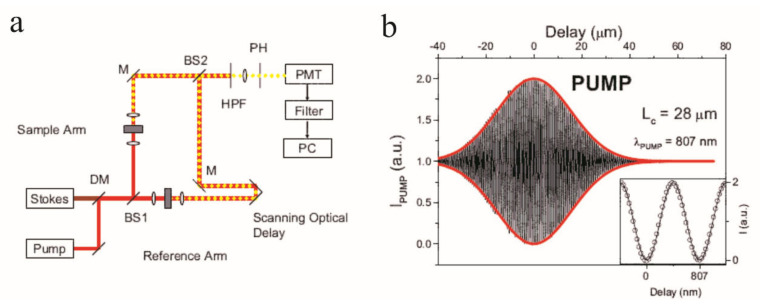
(**a**) Setup of the interferometric CARS measurement system. DM, dichroic mirror; BS, beamsplitter; M, mirror; HPF, high pass filter; PH, pinhole; PMT photomultiplier tube; PC, personal computer. (**b**) CARS interferogram detected at the beamsplitter BS2 of the setup shown in (**a**). In the inset is shown a detail of the interference pattern and its fit by the real part of the degree of coherence function (open circles: experimental data; solid line: fit). L_c_ is the coherence length of the pulse. λ_AS_ is the wavelength of the CARS signal. Adapted with permission from [61]. Copyright 2004 Optical Society of America.

Nonlinear interferometric vibrational imaging (NIVI) is a technique that utilizes coherent anti-Stokes Raman scattering (CARS) spectroscopy to simultaneously identify and locate molecules in the sample. CARS is a nonlinear four-wave mixing process that employs a pump, Stokes, and probe excitation fields to address designated molecular vibrations, and it generates stronger coherent signals than the spontaneous Raman emission [58]. Common CARS microscopy techniques count the anti-Stokes photons but ignore the spectral phase information, but NIVI takes advantage of the fast and sensitive spectral-domain detection to reconstruct a high resolution Raman spectrum while eliminating the background noise. In NIVI schemes, CARS signals are exploited in interferometer-based systems like OCT. Figure 6a shows a typical interferometric CARS measurement system.

Boppart et al., presented a NIVI technique for specific molecular species imaging in biological tissues using OCT [59,60]. This method splits the pump and Stokes beams, which are spatially and temporally overlapped in advance, into the two arms of an interferometer to generate reference and sample CARS signals at the same time. To generate strong reference signals, a target molecular species is scanned by the reference arm, and a sample is scanned by the probe arm. Both signals meet at the interferometer, and a stable spectral interferogram will be observed if the same molecular species exist in the sample. The CARS spectrum is considered as a signature of target molecules in a volume. In an experiment [61], the possibility to generate two anti-Stokes signals in two separate samples of benzene was proven, and the molecular-sensitive imaging capacity of NIVI was successfully demonstrated. Figure 6b illustrates the interference pattern of the detected CARS interferogram and it well fits the real part of the degree of coherence function. By switching between different molecular spices in the reference arm, NIVI can detect the corresponding molecules in the sample. However, in this scheme, the CARS signals are detected in the phase-matched forward collection because the signals are very weak. It is not suitable for highly scattering, non-transparent samples, such as biological tissues.

Later, Bredfeldt et al., demonstrated that a NIVI-based OCT system can distinguish CARS signals with a high vertical resolution [62]. In this experiment, a sandwich-like phantom was produced in which there are two 100-µm-thick layers of acetone with a thin layer of air in between. Different layers are separated by 150-µm-thick coverslips. In the pump, Stokes field, strong forward-directed CARS signals are generated in both layers of acetone and partly reflected at the acetone-glass interface behand. When the second layer of acetone is placed at the focus, the corresponding CARS signal has a greater amplitude than that from the first layer. It indicates the interferometric depth measurement capacity of the conventional OCT and NIVI-based OCT. CARS signals can be axially measured at over 0.6 mm depth of a sample with a low-numerical-aperture objective. Moreover, the first molecular imaging of a biological specimen, a thin layer of lipid-rich beef tissue compressed between two coverslips, was performed to depict the location of lipid.

NIVI-based OCT is a promising technique that combines the optical ranging technique of conventional OCT and the molecular sensitivity of CARS. With broadband Stokes pulse and narrowband pump/probe pulse, CARS can obtain multiplexed imaging of the contrast distributions of a wide range of molecular species. In addition to its capacity for label-free imaging, NIVI has other notable merits of good molecular sensitivity, efficiency, and temporal resolution. The excitation pulse energy varies depending on the application. It is a significant challenge that if a sample is highly scattering or the target molecules concentration is very low, the energy required for NIVI imaging is usually far beyond the ANSI suggested limitation for safety. In addition, the backscattered CARS signal is generally much weaker than forward-directed ones. As a result, NIVI is gradually becoming a successful microscopic technique that has been used in studies of animal tumor models and skin diseases [63,64,65,66]. In the future, the development of supercontinuum sources and epi-detection techniques may eventually lead to broader applicability of NIVI-based OCT to address in vivo biological studies and medical applications.

## 6. Second Harmonic OCT and Its Applications

Second harmonic OCT (SH-OCT) has a similar structure as NIVI-based OCT. It relies on the optical phenomena that second harmonic waves can generate if scanned samples contain noncentrosymmetric structures, such as chiral biological molecules in the biological tissues. In an SH-OCT system, the reference SH signals are produced through a nonlinear crystal in the reference arm. As the incident light to the sample and the reference arm comes from the same light source, the emitted SH signals are coherent and maintain a certain phase relationship with each other. Under certain conditions, they can be detected with the coherence interferometry in OCT [67]. The SH signal adds unique contrast enhancement to conventional OCT because it strongly depends on the polarization, orientation, and other local symmetry properties of the contrast agent. Therefore, SH-OCT has a similar axial resolution as conventional OCT but a higher lateral resolution due to the second harmonic signals [68,69].

**Figure 7:**
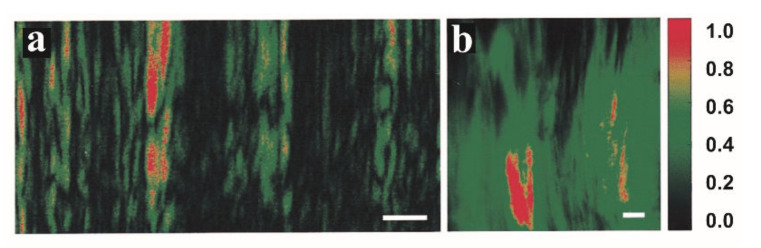
(**a**) SH-OCT image showing an area of 100 × 50 μm in the rat-tail tendon, where many cable-like, parallel oriented, and slightly wavy collagen fiber bundles (fascicles) can be visualized; (**b**) 60× polarization microscope image of the same sample (scale bar: 10 μm). Adapted with permission from [71]. Copyright 2005 AIP Publishing LLC.

Jiang et al., presented the first SH-OCT system using a femtosecond laser centered at 800 nm as the light source [70]. A polarizing splitter separated the input beam into two beams before they went in the two arms of the interferometer in OCT. The reference SH wave at 400 nm was generated through a nonlinear crystal of β-barium borate at the reference arm. A verification of this SH-OCT scheme was carried out using a polished GaAs crystal instead of a sample to produce surface SH signals. The interference of the original probe waves and the SH waves was measured simultaneously. Once proved that the experimental values accord well with the theoretical values, the developers acquired SH-OCT images of two 30-µm-thick collagen layers between three 170-µm-thick coverslips. Because collagen in the biological tissue has a triple helical structure, it is an efficient source of SH generation [70,71,72]. The average excitation power was about 50 mW and the peak power density in the sample was about 3.2 GW/cm^2^. Conventional OCT signals were observed at all interfaces, but SH-OCT signals only appeared at two collagen–glass interfaces. The axial resolution of their second SH-OCT system is sixfold higher than the previous one [71]. A photodiode and a photomultiplier were employed to detect the conventional OCT signals and the SH-OCT signals, respectively. The SH-OCT image of a rat-tail tendon with a transverse and axial resolution of 1.9 and 4.2 µm was demonstrated in Figure 7a. The collagen fibrillar matrix structure of the rat-tail tendon was revealed clearly, which matched the result from the microscope (Figure 7b). However, the average power was up to 80 mW. The same group also implemented an ultra-wideband photonic crystal fiber to deal with the original excitation wave and the SH wave [73]. It ranges from 400 nm to 1400 nm which is especially suitable for SH-OCT schemes. To increase the imaging speed, they developed a frequency-domain SH-OCT to replace the time-domain SH-OCT setup [74]. The new system is more than two orders of magnitude faster than previous systems. However, its axial resolution is decreased to 12 µm because the new system requires a broadband light source to maintain the imaging resolution. The SH-OCT images of several biological samples including fish scales, pig leg tendon, and rabbit eye sclera were demonstrated successfully. Two other groups also presented their SH-OCT systems, respectively, [61,68,75]. It is worth noting that Applegate et al., demonstrated the first polarization-resolved SH-OCT system on a salmon belly skin sample with molecular-specific and polarization-resolved images [75]. The structures rich in collagen and the relative orientation of collagen fibers are clearly depicted at an ultrahigh axial resolution (<5 μm). Later, they presented their frequency-domain SH-OCT as well [68].

The SH-OCT technique has numerous advantages. First, SH-OCT requires minor changes in the hardware and software of conventional OCT. Second, the coherence gating control of OCT can assist SH-OCT to achieve a high axial resolution and enhanced SH contrast deep inside the sample. Besides, SH signals do not require staining of the sample. In biological tissues, collagen is the primary endogenous contrast agent for SH-OCT imaging. As collagen modifications are found in various physiologic processes, SH-OCT should be easily extended to applications such as wound skin healing, aging, diabetes, and cancer [76,77]. Up to now, there is no reported exogenous contrast agent for SH-OCT imaging. In theory, any big molecules having unsymmetrical structure and nonlinear polarizability could be candidates as SH-OCT contrast agents, such as bacteriorhodopsin [78,79]. Moreover, the development of new materials may also contribute to finding new contrast agents [80,81,82]. Although the SH generation is the most efficient and accessible nonlinear light generation process that requires relatively low excitation energy, its light intensity is still above the ANSI limitation. By far, SH generation is mostly used as a microscopic technique [83,84,85].

## 7. Magnetomotive OCT and Its Applications

Magnetomotive OCT (MM-OCT) generates specific and sensitive molecular images using external magnetic fields and magnetic exogenous contrast agents [86]. In this method, magnetic contrast agents are introduced into the sample before the scanning. Before the scanning, an additional conventional OCT image of the sample is acquired as the background signal. Next, the MM-OCT applies a modulated magnetic field to the sample. Under the modulated magnetic force and the viscoelastic restoring force from the surrounding tissue, magnetic nanoparticles will have nanoscale movements along with the deformation of the elastic, such as shifting in and out of the OCT probe beam or rotating in place. The movements result in the interference signals of OCT. By subtracting the background signals, the researchers calculated the amplitude of interference signals, which revealed the distribution of contrast agents.

Toublan et al., discussed the potential use of magnetically inducible optical contrast agents to produce molecular contrast signals during the OCT scanning [87]. Next year, the same team implemented the original MM-OCT system and demonstrated the first MM-OCT image of macrophages labeled using phagocytosed hematite particles [88,89]. Later, they demonstrated the first in vivo MM-OCT image for the digestive tract of an African clawed frog tadpole with 220 µg/g magnetite nanoparticles that matches the actual histology [90]. They also studied the impact of the magnetic field on the nanoparticle distribution and the corresponding changes in the image intensity, penetration, and scattering properties.

Oldenburg et al., presented the first modern MM-OCT system which achieves the deployment of magnetic contrast agents by measuring their periodic phase shift with conventional or phase-sensitive OCT, instead of tracking the interference signals [91,92]. The new system is able to measure the diffusion of magnetic nanoparticles (MNPs) into real biological tissue samples: excised rat mammary tumors. The tumor sample was immersed in a solution with polymer-coated magnetite/maghemite nanoparticles (~20 nm) for 15 min. During the scan, a magnetic field of 0.08 T was modulated at 55.6 Hz. The modulation frequency of the magnetic field was set to beyond the OCT frequency in order to separate the magnetomotive and optical scattering signals into two channels. The dynamic range of MM-OCT images was about 16 dB. It brought tenfold improvement of the signal quality and imaging speed than the original design. Since then, many attempts have been made to improve the MM-OCT design. Kim et al., developed dual-coil MM-OCT for liquid sample scanning [93]. In 2016, they developed a metal-free intravascular MM-OCT system to scan diseased atherosclerotic rabbit aortas with polyethylene glycol-covered iron oxides particles [94]. Both early stage fatty streaks and early stage atherosclerotic lesions were labeled with αvβ3 integrin-targeted magnetic nanoparticles and were revealed by the new MM-OCT system. With its molecularly sensitive and dynamic contrast, MM-OCT can be used for elastography as well. Ma et al., presented a modulation noise reduction design with a common-path-based device to increase the MM-OCT sensitivity 20 times over [95].

As biological tissues usually exhibit a diamagnetic response, they need exogenous contrast agents to produce MM-OCT signals. Novel contrast agent development has become a hot point of the MM-OCT study. MNPs composed of biocompatible iron oxides (magnetite or maghemite) are the most common sources of MM-OCT contrast agents [91]. Indoliya et al., synthesized a series of superparamagnetic iron oxide nanoparticles (SPIONs) as multimodal contrast agents for both MM-OCT and PT-OCT [86]. To improve the hydrophilia and biocompatibility of the SPIONs, they were coated with polyethylene glycol. The average size is 2.28 nm for bare nanoparticles and 16.44 nm for polymer-coated particles. After applying the SPIONs to the dorsal part skin of live swiss albino mice, they were scanned in vivo with in-house built MM-OCT and PT-OCT. The magnetic field of MM-OCT is 0.086 T and was operated at 30 Hz frequency during the scan. Compared with standard OCT images, the contrast of MM-OCT images is enhanced. The magnetomotive signal also delineated the presence and distribution of SPIONs inside the tissue. Meanwhile, these SPIONs also enhanced the PT-OCT images of the skin of mice with an extra 980 nm pump laser. The diffusion dynamics of both coated and bare SPIONs inside the mouse skin were studied using MM-OCT and PT-OCT side-by-side. It is found that the nanoparticles enhance the OCT contrast while gradually penetrating into the tissue. The polymer-coated SPIONs show a better diffusion speed and penetration ability than bare SPIONs.

**Figure 8:**
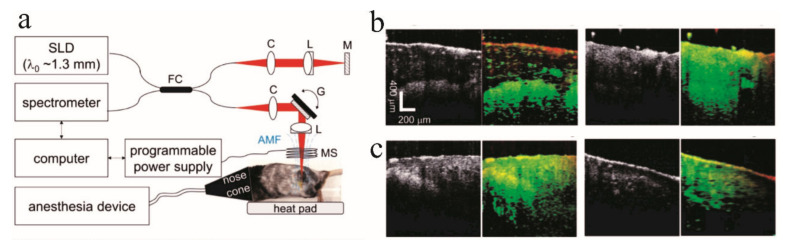
(**a**) Experimental flowchart of MM-OCT imaging of in vivo MH-treated melanoma-bearing mice. (**b**) Simultaneously acquired in vivo OCT (**left**) and MM-OCT (**right**) depth-resolved cross-sectional images of melanoma tumor tissue with high overall and local cellularity, (**c**) low overall and local cellularity. Adapted with permission from [96]. Copyright 2021 The author(s).

Huang et al., exploited the theranostic functionality of dextran-coated iron oxide nanoparticles through their concentration and displacement from in vivo MM-OCT images of melanoma-bearing mice [96]. Maghemite Fe_2_O_3_ and dextran were mixed in sodium hydroxide (NaOH) solution to synthesize these nanoparticles. A nanoparticle solution was injected into the melanoma tumor of mice, and the injection sites were scanned with MM-OCT before and after the magnetic nanoparticle hyperthermia therapy as Figure 8 presents. The depth-resolved 2D images of in vivo OCT and MM-OCT provide the structural and molecular information of the injection sites, respectively. In this case, these nanoparticles are both contrast agents and therapeutic agents so that MM-OCT can validate the nanoparticle delivery and the treatment effect simultaneously. Similarly, Wijesinghe et al., synthesized a type of amino-polyvinyl alcohol coated SPIONs (80–150 nm size) for tumor imaging [97]. New contrast agents were tested in pork belly samples and in vivo melanoma skin cancer samples of mice. It was found that the nanoparticle motion can be traced continuously in MM-OCT at high accuracy.

As noted above, MM-OCT is a powerful tool for morphological, biomechanical, and molecular biology studies, which can provide sensitive, high background-rejection images of MNPs in biological tissues. The extra magnet field is generally regarded as safe for humans. Another advantage of MM-OCT is that it can use MRI contrast agents, in which SPIONs have been approved by FDA. The development of novel magnetomotive contrast agents is relatively easy [98,99,100]. Besides, many synthesized magnetic microspheres have been developed as efficient contrast agents for multiple imaging modalities, for instance, MM-OCT and fluorescence [101]. Such a multimodal imaging system can provide both large fields of view and cellular resolution. However, the safety of the MNPs used for MM-OCT has not been fully investigated. The imaging speed of MM-OCT can be improved by using SD-OCT system, but it is fundamentally limited by the modulation frequency of the external magnetic fields, which is restricted by the biological tissue mechanics.

## 8. Photothermal OCT and Its Applications

Photothermal OCT (PT-OCT) is a functional OCT technique that measures the distribution of optical absorbers in scattering samples. It takes advantage of the photothermal response, where the chromophores absorb the energy of photons and result in a rise in local temperature. During a PT-OCT scan, an extra laser beam is coupled to the conventional phase-sensitive OCT, which goes through the probing arm to heat the sample. The increase in temperature leads to thermal expansion surrounding the contrast agents and changes of the refractive index of the target region, both of which result in changes in optical path length. The path length changes are usually at a nanometer level and can be detected as shifts in the phase signals. Therefore, PT-OCT can actively identify the absorbers in the sample with high sensitivity and specificity. To facilitate the generation of PT-OCT signals, the photothermal laser beam is amplitude-modulated at a certain frequency for the intermittent heating and cooling of the sample. Repeated A-scans are applied to detect thermal cycles for every sample location. In the presence of the contrast agents, shifts at the same frequency of laser modulation can be observed over time, and the average amplitude of these shifts is recorded as the PT-OCT signal at this location. There are many parameters that affect the amplitude of shifts, but the PT-OCT signal strength is proportional to the number of absorbers in the sample when keeping all other parameters constant.

Previous studies explored the photothermal imaging of nanoparticles that combines heating modulation and interference contrast [102], but Fujimoto et al., presented the first modern PT-OCT system [103]. They applied modulated 808 nm photothermal laser to gold nanoshells in deionized water and recorded obvious regular PT-OCT signals. By contrast, no PT-OCT signal was found in absence of nanoshells or photothermal lasers. In the optimal SNR condition, the temperature rise caused by the photothermal laser is about 0.52 °C, which should be harmless to human tissues. In this study, the phantom was designed to isolate any effect other than absorption. However, in biological tissue samples, properties like scattering are as critical as absorption. Another team demonstrated the PT-OCT signal of gold nanospheres in tissue-like scattering phantoms in the same year [104]. Moreover, the nanospheres were functionalized to target cells that overexpress epidermal growth factor receptor (ECFR). Validation results displayed three times higher PT-OCT signals in cells having the highest amount of EGFR (MDA-MB-468) than cells expressing low levels of DGFR (MDA-MB-435).

Paranjape et al., published an application to detect macrophages in ex vivo rabbit artery plaques using PT-OCT [105]. It is the first time to measure the photothermal-generated shifts in both surface and inside of the tissue. Gold-coated iron oxide (nanorose) particles were used as the contrast agents. Artery tissues with nanoroses exhibited stronger PT-OCT signals than pure tissue samples. Applications of PT-OCT to detect gold nanoshells in ex vivo human breast tissues were demonstrated as well [106]. Tucker-Schwartz et al., reported the first in vivo imaging of PT-OCT with gold nanorods that injected into the right ear of several mice. Experimental results showed a significant enhancement in signal in presence of gold nanorods than controls, which illustrated the potential of PT-OCT to be an in vivo, pre-clinical molecular imaging tool [107].

A main disadvantage of PT-OCT is that its imaging speed is significantly lower than the conventional OCT because the point-by-point thermal activation and subsequent temporal sampling are very time-consuming. Salimi et al., implemented a high-speed transient-mode PT-OCT that checks the transient thermal response with a single diode laser pulse [108,109]. Its effective A-line rate in multilayered samples is at least 100 times higher than before. Wu et al., developed a pulse PT-OCT system that separates the photothermal phase and Doppler phase synchronously for the first time [110]. The new scheme utilizes an even number differential demodulation algorithm to extract information for the quantitative characterization of biological indicators.

Because human tissues only contain a few effective light endogenous absorbers, many nanomaterials have been developed as contrast agents for PT-OCT scans. At first, in vitro and ex vivo research with targeted gold nanospheres [104], non-targeted gold nanoshells [103,106], gold nanorods [111,112], gold nanoroses [105], and carbon nanotubes [113]. These nanoparticles showed little signal in standard OCT scans but clearly showed their distribution in photothermal OCT scans. With the previous success, in vivo research has come to the fore. Tucker-Schwartz et al., injected gold nanorods into the right ear of several mice for the first in vivo imaging [107]. The size of gold nanorods was 45.2 ± 5.7 nm long by 13.2 ± 1.8 nm. Mohan et al., synthesized novel hydrophilic and biocompatible upconversion nanoparticles from NaGdY_4_ as a contrast agent for PT-OCT [114]. New nanoparticles are hexagonal phase, rod-shaped structures with an average size between 50 and 200 nm. Ex vivo imaging test of animal tissue phantom demonstrated that the new contrast agent could provide better image quality and extra contrast information.

**Figure 9:**
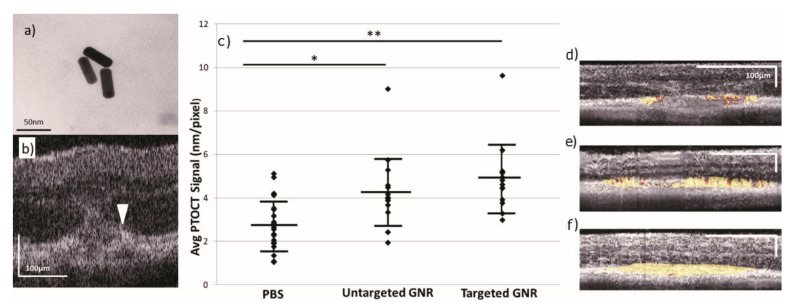
(**a**) Transmission electron micrographs of GNR demonstrating unaltered morphology following surface functionalization. (**b**) OCT in vivo image of day 5 laser-induced choroidal neovascularization lesions with disrupted RPE (white arrowhead). (**c**) PTOCT of untargeted and targeted GNR in vivo. Average PTOCT signal density for each cohort, with error bars representing standard error of the mean. There is a significant increase in this signal associated with both untargeted and targeted GNR injections versus PBS control. * *p* ≤ 0.05; ** *p* ≤ 0.001. (**d**–**f**) Representative OCT B-scans of mice injected with PBS (b; *n* = 21 eyes), untargeted GNR (c; *n* = 14 eyes), and targeted GNR (d; *n* = 14 eyes), respectively, with lesion-associated photothermal signal overlaid in gold. Note the increased concentration of photothermal signal associated with passive accumulation of GNR in the lesions, and the greater increase associated with the injection of targeted GNR. Adapted with permission from [116]. Copyright 2019 The author(s).

PT-OCT can be particularly effective at retinal disease imaging. Andrew et al., demonstrated PT-OCT in eyes for the first time as an in vivo noninvasive imaging approach to depict both endogenous melanin and exogenous gold nanorods [115]. Mice model with laser-induced choroidal neovascularization was prepared and injected carboxyl-functionalized polyethylene glycol (PEG)-coated-gold nanorods (10 nm in diameter and 35 nm in length) with a peak absorption of 750 nm via the tail vein. PT-OCT showed an increased signal due to the untargeted nanoparticle accumulation in the lesion area. Two years later, they also validated the performance of novel targeted gold nanorods (Figure 9a) as a PT-OCT contrast agent in the eye [116]. The photothermal signals from untargeted nanorods are about 1.49 times higher than the baseline, and the signals from targeted nanorods are 1.73 times higher than the baseline as Figure 9b demonstrates. The PT-OCT B-scans of mice injected with targeted nanorods showed stronger PT-OCT signals in the lesion region than that from the untargeted nanorods group. Examples are demonstrated in Figure 9d–f. The photothermal laser power can raise the average photothermal signal as well. The same research group also confirmed the high-specificity of indocyanine green, the only FDA-approved contrast agent in the eye, as a PT-OCT contrast agent in ex vivo pig eyes [117].

In conclusion, the PT-OCT technique is a promising method to enable molecular imaging that combines spatial resolution and imaging depth. There are however limitations of PT-OCT. First of all, to comply with the ANSI limitations for human skin and eyes, the photothermal laser power should be reduced in the future while achieving clear PT-OCT signals. Second, due to the Brownian movement, it is hard to measure phase shifts accurately inside the liquid than at the interface of the liquid samples. Mathematical models of the temperature increase induced by photothermal response have been derived, but it is still challenging to measure the temperature change at different depths. In the end, the imaging speed of PT-OCT is not yet sufficient for the applications requiring fast scans.

## 9. Spectroscopic OCT and Its Applications

The absorption spectrum has been widely used to identify specific molecules. The low coherence light source of OCT has a broad bandwidth which is especially suitable for absorption spectroscopy. Based on the spectral analysis, spectroscopic OCT (S-OCT) can yield information about the properties of chromophores through the absorption contrast of backscattered light from various depths of the sample and thus depict tissue pathologies or functional states. Compared with conventional spectroscopy, S-OCT allows quantitative spectroscopy in a certain location without disturbance from the surrounding tissue. An S-OCT system has a tradeoff between the axial and the spectral resolution because a high spectral resolution will naturally reduce the axial resolution during the short-time Fourier transformation [118]. Contrast agents can enhance the spectroscopic signal, and they usually have absorption spectrum peaks in the wavelength region of OCT light sources. If the absorption peak of a contrast agent mismatches the center wavelength of the OCT light, the contrast agent in the sample will absorb part of the probe light spectrum and lead to spectrum changes of the backscattered light. By analyzing the interferometric OCT signal, S-OCT can uncover local scattering and absorption and consequently reveal the existence of contrast agents in the tissue.

**Figure 10:**
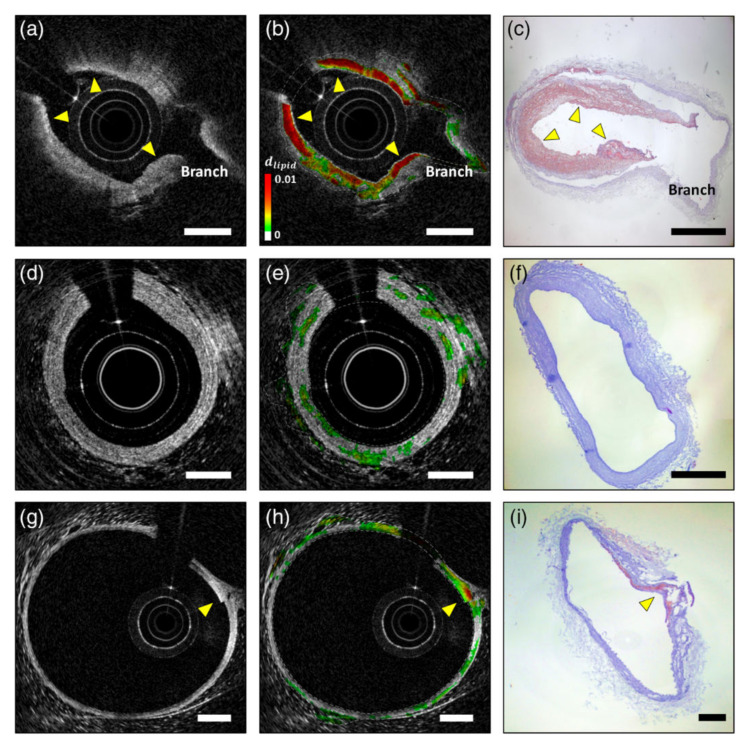
Representative results of mapping the lipid distribution function and comparison with the corresponding histology sections. The first, second, and third rows show the results for lipid-rich plaque (**a**–**c**), fibrous plaque (**d**–**f**), and no lesion (**g**–**i**), respectively. Gray-scale OCT images (**a**,**d**,**g**) only provide morphological information, whereas the mapping results of the lipid distribution function derived from S-OCT (**b**,**e**,**h**) provide information about lipid composition, which corresponded well with ORO-stained histological sections (**c**,**f**,**i**). Scale bars, 500 µm. Adapted with permission from [128]. Copyright 2016 SPIE.

Hemoglobin is the major endogenous contrast agent for S-OCT, while NIR dyes and nanoparticles are two main categories of exogenous S-OCT contrast agents. Morgner et al., developed the first S-OCT device with probe light bandwidth of 230 nm and center wavelength at 800 nm [119]. Its depth and lateral resolution are about 1 µm and 5 µm, respectively. This S-OCT system detected the whole bandwidth of the available spectrum in one measurement and enhanced the image contrast of a *Xenopus laevis* tadpole without any exogenous contrast agents during the demonstration. Xu et al., gave an exhaustive discussion about the common NIR dyes suitable for S-OCT scan and demonstrated that NIR dyes can work along well with other nanoparticles as contrast agents. For example, NIR dyes mixed with 100 nm nanospheres [120]. NIR dyes can also be encapsulated in protein nanospheres as OCT contrast agents [18]. Most of the S-OCT are software-based, but Yang et al., developed a novel S-OCT hardware to detect molecules by triangulating a peak in the absorption spectra with three different light sources [121]. They used this device to map the distribution of indocyanine green in *Xenopus laevis*. Xu et al., also pointed out that plasmon resonant nanoparticle contrast agents with high extinction coefficients could be detected using S-OCT. In general, particles larger than 100 nm are dominated by resonant scattering effects, whereas particles smaller than 100 nm exhibit absorption. Oldenburg et al., presented a type of gold nanorods as a contrast agent, which is 50 nm long with an aspect ratio of 3 [122,123]. Its plasmon-resonance peak is 755 nm and overlaps the OCT light wavelength band. The contrast agents enable S-OCT to achieve clear images with low noise in excised human breast carcinoma. Xingde Li et al., presented a series of research on the gold nanocages as contrast agents of S-OCT [124,125]. Gold nanocages with an edge length of 35 nm were produced, which have a tunable plasmon resonance peak in the NIR region. Experiments showed that gold nanocages have five orders of magnitude larger absorption ability than common dyes in tissue phantoms. On that basis, the first gold nanocage-based scattering-dominant agent was synthesized and validated for in vivo tumor imaging, proving significant contrast enhancement. Spicer et al., calibrate the system spectral response of an S-OCT system with a solution of 80 nm polystyrene beads in water [126]. Si et al., also used PEGylated gold nanobipyramids as contrast agents for multiplexing in a tumor-implanted mouse model. In vivo imaging of mouse pinnae showed that the spectral contrast in the tissue increases linearly for nanoparticle concentration [127]. Nam et al., reported an algorithm to characterize lipid plaques with S-OCT based on a Gaussian center of mass (GCOM) metric [128]. Using an atherosclerotic rabbit model, they demonstrated the in vivo S-OCT images of lipid-rich plaques as Figure 10 shows. The plaques detected using S-OCT match well with ORO-stained histological sections, and the validation results indicate that this method has a sensitivity and specificity of 94.3% and 76.7% for lipid detection, respectively.

The S-OCT technique has been confirmed to have the potential for microstructure evaluation at the nanoscale level [119,126,129,130]. Martin Leahy et al., presented an S-OCT method to achieve nanoscale measurements with spectroscopic contrast [12]. It transforms spectral amplitudes to axial spatial frequency amplitudes for sub-bands decomposition. The spatial period profile of a given point in the sample is calculated based on the sub-bands energy distribution. This method enables a conventional OCT with a resolution of 30 μm × 30 μm × 12 μm to detect about 30 nm size difference between two layers of nanospheres. Its sensitivity is more than 300 times higher than before. Therefore, it may monitor the movements or changes of nanostructures. Years later, they assessed wound healing within the cornea with this method [15].

Currently, S-OCT benefits from the fast development of nanotechnology. Many nanoparticles, such as gold nanorods, gold nanoshells, and gold nanocages, have been validated as optically and chemically stable S-OCT contrast agents for widespread applications in biomedical fields. At the same time, extensions of S-OCT such as spectroscopic optical coherence refraction tomography (SOCRT) have been developed. SOCRT reconstructs spectroscopic images from S-OCT scans at different angles for higher spatial resolution, showing excellent potential to measure nanostructures [131]. As the S-OCT signal is computed by taking the bandwidth of OCT light and dividing it using the number of pixels for every A-scan, broader bandwidth light sources can improve the resolution of S-OCT. Thus, using visible light instead of NIR light in S-OCT is potentially a better way to identify molecules in biological tissues.

## 10. Polarization-Sensitive OCT and Its Applications

Polarization-sensitive OCT (PS-OCT) is the first developed functional extension of conventional OCT. It acquires polarization properties of a sample with just a few modifications to the standard OCT framework. Based on the reflected or backscattered light, PS-OCT can quantitatively measure the polarization state for every pixel or voxel in an axial scanning [132]. Since polarization states can reveal ultrastructure changes below the optical resolution level of standard OCT, PS-OCT has been applied in many biomedical fields. There are four major polarization effects that can affect the state of a probe beam when it interacts with a sample: preserved polarization, birefringence, diattenuation, and depolarization. Several PS-OCT approaches have been designed to measure these polarization effects. Hee et al., presented the scheme of the first PS-OCT system with a single circular input state [133]. Modern PS-OCT designs can access the Jones matrix characterization, Stokes vector quantification, and Müller matrix measurements based on multiple polarization state inputs [134]. Jones matrix is composed of eight variables and can describe any polarization states other than depolarization. Since Jones vectors describe pure polarization states, Stokes vectors and Müller matrix are used to describe depolarization and partially polarization states. As only the linear birefringence and diattenuation properties of a sample can be detected directly, the degree of polarization uniformity (DOPU) was defined to measure more polarization properties. Therefore, PS-OCT can quantitate the polarization states of biological tissue samples, though it is not a typical molecular imaging method.

PS-OCT is a valuable tool to measure both the size and shape of nanoscale structures, such as nanoparticles. Schneider et al., used a PS-OCT to measure nanoparticles and avoided its performance with spherical and disk-like particles [135]. They simulated the particle size and shape from their scattering parameters. The results suggested that spherical particles tend to maintain the polarization state of the scanning light, while non-spherical particles are likely to reflect light with changed polarization states. In the validation, both the size and shape of different nanospheres and disk-like nano-clay particles were successfully achieved with simulations. They also found strong cross-polarization backscattering from TiO_2_ nanoparticles in polyamide 66. Lippok et al., presented the first method to demonstrate the diffusion and concentration of gold nanorods from their depolarization signatures with PS-OCT [136]. A series of clinically and biologically relevant experiments were designed for ex vivo, in vitro, and in vivo validation. The sizes of gold nanorods in experiments are 10 × 81 nm and 10 × 67 nm. This method may contribute to the image quality by suppressing artifacts and simplifying some stringent experiments. Strakowska et al., presented a non-destructive way to assess the characterization of thin hydroxyapatite layers outside metal particles with PS-OCT [137]. The hydroxyapatite coat was synthesized outside silver nanoparticles using the sol-gel method, which suggests that the PS-OCT technique is a valuable method for nanostructure evaluation.

**Figure 11:**
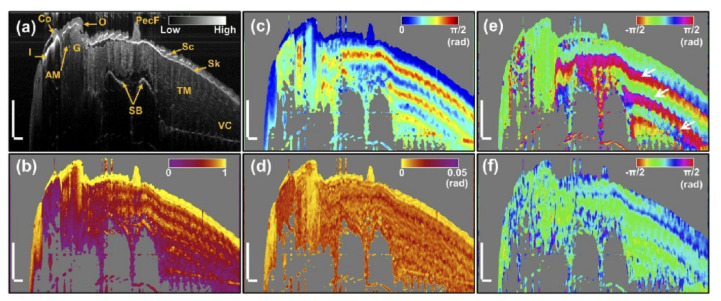
PS-OCT sagittal images of the zebrafish. (**a**–**f**) Intensity, degree of polarization uniformity, accumulative retardation, local retardation, accumulative optic axis, and local optic axis image, respectively. The iris (I), cornea (Co), adductor mandibulae (AM), opercle (O), gill (G), pectoral fin (PecF), scales (Sc), skin (Sk), trunk musculature (TM), swim bladder (SB) and vertebral column (VC) can be identified in (**a**). Scale bars are 500 μm. Adapted with permission from [143]. Copyright 2020 Optical Society of America.

Many biological tissues exhibit strong birefringence and depolarization effects, and therefore they can be easily separated from the surrounding environment [138]. For example, fibrous tissues, collagen, dental enamel, and nerve fibers have the birefringence effect, and melanin pigments exhibit the depolarization effect. Sugita et al., traced retinal nerve fiber bundle with PS-OCT, in which Stokes vector averaging with re-normalization is applied for polarization state averaging [139]. Nerve fibers in cerebral white matter or peripheral nerves can be demonstrated in PS-OCT images as well [140]. PS-OCT also can detect macular degeneration based on the depolarization in pigmented structures [141]. Tendons and muscles have strong birefringence because they are rich in collagen. De Boer et al., demonstrated the tendon by PS-OCT for the first time [142]. Yang et al., distinguished different muscles of zebrafish by extracting polarization information from PS-OCT images as Figure 11 illustrates [143]. It suggests that PS-OCT can provide abundant function information other than structure information. There are many reported applications of PS-OCT including ophthalmology [139,144,145,146,147], cardiology [148,149,150,151], dermatology [152,153,154,155], odontology [156,157,158] and oncology [159,160,161,162].

Another series of exciting applications of PS-OCT is scanning with exogenous contrast agents, especially gold nanoparticles which have a strong depolarization effect [136,163,164]. PS-OCT is capable of measuring their dynamic scattering signals and polarization properties. Lippok et al., demonstrated the distribution of gold nanorods ex vivo, in vitro, and in vivo according to the depolarization [136]. The relation between the degree of depolarization and the nanoparticle concentration was analyzed during the validation. Chhetri et al., demonstrated the potential of PS-OCT to probe biological nanotopological changes with depth-resolved images of diffused gold nanoparticles in pulmonary mucus and mammary extracellular matrix [165]. Nanoscale viscoelastic properties studies have benefited from the plasmon-resonant and highly anisotropic properties of gold nanoparticles. Keahey et al., also demonstrated that gold nanobipyramids have distinguished signatures in lymphatic vessels and blood vessels of live mice [164].

With high imaging speed and sensitivity, the PS-OCT technique has been applied to many fields. By measuring the polarization states of a sample, it provides unique tissue-specific contrast for quantitative measurements. The polarization properties can be analyzed in several mathematical frameworks: the Jones formalism, the Stokes formalism, and Müller formalism. The main shortcoming of Jones’s formalism is that it cannot describe partially polarized light and depolarization effects. Such weaknesses have been addressed by Stokes parameters and Müller matrix, but it requires repeated scanning for the same location to determine the Müller matrix completely, which significantly increases the scanning speed. PS-OCT is a valuable tool for biological tissue analysis. Fibrous tissues like muscle, nerve fiber, and collagen-rich tissue exhibit birefringence effects. Melanin granules in the skin or iris can be detected due to their depolarization effects. Exogenous scattering particles such as gold nanoparticles can lead to depolarization as well. In the future, the potentially important applications of PS-OCT include tumor tissue delineation and drug delivery tracking.

## 11. Discussion and Outlook

### 11.1. Molecular Contrast OCT Techniques

The molecular contrast OCT techniques highlighted in this review can be categorized into three broad groups. The first group relies on the light scattering and/or absorption of certain molecules in the sample, which includes the engineered particle-based method, PP-OCT, pump suppression OCT, S-OCT, and PS-OCT. Both PP-OCT and pump suppression OCT extract the distribution of contrast agents based on their changed absorption spectrum. The engineered particle-based method utilizes both the scattering and the absorption variation introduced by contrast agents to map their location. S-OCT reveals the specific tissue properties from the spectral variation of absorption. PS-OCT measures the polarization state changes of the backscattered light to confirm the existence of certain tissues. The second group acquires molecular information by generating and detecting coherent light from certain molecules in the sample. SH-OCT and NIVI produce coherent emissions with the second harmonic process and the coherent anti-Stokes Raman scattering process respectively. These coherent signals are detectable using interferometric methods. The last group contains PT-OCT and MM-OCT, which introduce modulated phase shifts into the pathway of the backscattered light to exhibit the distribution of contrast agents.

In consideration of safety profile, among all the presented approaches, the engineered particle-based method, S-OCT and PS-OCT passively interrogate the existence and distribution of molecular contrast agents. These methods require no extra energy but only optical component upgrades of conventional OCT systems, which are safe and relatively simple. MM-OCT imaging requires an extra modulated magnetic field, but it should be safe as well. By comparison, PP-OCT, pump suppression OCT, SH-OCT, NIVI, and PT-OCT need to introduce additional light pluses for optical excitation or thermal effect. The light fluences onto the sample are usually high and raise the concern about the possible photoinduced damage. Compared with the ANSI recommend limits [166], almost all molecular contrast OCT approaches work within the guideline, except SH-OCT and NIVI.

There are significant differences between the imaging speed of different modalities. PP-OCT, pump suppression OCT, PT-OCT, and MM-OCT rely on time-encoded molecular signals from extra modulated energy fields. These methods require multiple A-lines at the same position to calculate the molecular signals, which will significantly lower the imaging speed. In contrast, the imaging speed of the other molecular OCT imaging methods is almost as fast as conventional OCT.

The image quality also varies between these techniques. A method with a high signal-to-noise ratio (SNR) suggests that it can detect low concentrations of contrast agents. To generate one image, PP-OCT and pump suppression OCT need to scan before and after the light excitation. The time lag between these two scans is relative long. This step will cause speckle noise and decrease the SNR. Pump suppression OCT is more affected than PP-OCT due to its long interval. The image quality of SH-OCT and NIVI is mainly affected by the concentration of targeted molecules, the bandwidth of the incident light, and the epi-detection technique. In comparison, S-OCT requires a balance between image resolution and molecular contrast resolution, which needs an extra system hardware upgrade to solve this dilemma. PT-OCT and MM-OCT measure molecular signals from the pathway changes of the backscattered probe light. These changes are so small that the resultant signals are comparable to the system noise. However, this problem can largely be addressed with additional signal processing algorithms and system hardware. In general, all modulation methods are expected to be less sensitive to the background noise in biological tissues since the changes exhibit a specific modulation frequency outside the baseband. It should be noted that though the photothermal effect is the key for PT-OCT imaging, excessive energy would lower the image resolution by affecting the tissue around the target region. The features of the presented molecular contrast OCT techniques in this review are summarized in Table 2.

### 11.2. Molecular Contrast Agents

Both endogenous and exogenous contrast agents have been widely used for molecular contrast OCT techniques to label and visualize specific molecules in biological tissues. There are far more kinds of extrinsic contrast agents than intrinsic ones, which include dyes, nanoparticles, gas vesicles, and so on. An exogenous contrast agent is targeted if it only strengthens the molecular contrast of specific regions. On the contrary, an untargeted contrast agent will enhance the image contrast of a sample in general. It means that the molecular contrast from imaging agents within the biological tissues could be confounded by the background scattering and speckles from the samples. Although most of the reported contrast agents of molecular contrast OCT are still untargeted so far, some types of contrast agents like GNSs and nanoparticles with protein or poly shells can be functionalized to target specific cell sites with relative ease. Among them, plasmonic gold nanoparticles are of particular interest as they have good biocompatibility, highly tunable size, shape, and optical properties. Despite these promising features, the potential use of gold nanoparticles for molecular contrast OCT imaging has not been fully explored. Actually, for any potential molecular contrast agent, its characteristics such as synthesis, stability, toxicity, biocompatibility, and biodegradability should be thoroughly researched, as this would make up the first step towards clinical applications. Every molecular contrast OCT technique has its own choices of molecular contrast agents. The typical contrast agents for molecular contrast OCT techniques are given in Table 3.

The reported exogenous contrast agents for PP-OCT include indocyanine green [45], DsRed [43], and methylene blue [50,51,52,53]. Both indocyanine green and methylene blue are FDA-approved contrast agents. Obviously, endogenous or FDA-approved contrast agents are able to prompt the corresponding molecular contrast OCT techniques to clinical applications. Dyes with absorption bands in the NIR regime, such as methylene blue and indocyanine green, have the potential to label multiple molecules. However, it is still a challenge to control the concentration and distribution of these dyes. Combining dyes with nanotechnology for better contrast agents is a promising trend. For example, biodegradable PLGA encapsulated dyes particles have good penetration performance with a remarkable reduction of the singlet oxygen generation from the pump excitation [51,52,53]. Besides, PLGA shells can be functionalized with ligands for targeting molecular biomarkers [53]. Another advantage of PLGA is that it is FDA-approved for drug delivery and tissue engineering applications.

Pump suppression OCT has only two reported molecular contrast agents, namely bacteriorhodopsin [167] and phytochrome A [168,169]. New species of contrast agents are still highly desired. Similar situations occur with contrast agents for molecular imaging of SH-OCT, as only collagen has been used so far. It is because an SH-OCT contrast agent should have a high endogenous SH generation coefficient and can be well aligned in the sample. Given the specific advantages of SH-OCT, the development of new contrast agents would be highly rewarded.

Benzene, acetone, and lipid were used as molecular contrast agents for NIVI scanning in the presented studies. In theory, NIVI could detect any molecules in the sample by using the corresponding molecular spices in the reference arm. However, it requires powerful excitation energy to produce images with acceptable quality in situations like highly scattering samples and low target molecule concentrations. In the future, finding the contrast agents with high sensitivity for NIVI would be the first step toward NIVI applications.

MM-OCT has only extrinsic molecular contrast agents, most of which are MNPs. MNPs are designed for MRI imaging initially and have been used for MM-OCT imaging. Among all MNPs, SPIONs are particularly attractive due to their excellent magnetic properties, biocompatibility, and biodegradability [170]. The degradation product of MNPs, ferric iron, is considered safe because it can participate in normal metabolism. However, the degrees of MNPs’ cytotoxicity and genotoxicity are still under investigation [171]. So far, SPIONs are FDA-approved MRI contrast agents and therefore may benefit the MM-OCT clinical applications. Some types of MNPs can be modified as targeted contrast agents and used as photothermal OCT contrast agents at the same time.

There is a wide range of PT-OCT contrast agents. It contains a list of intrinsic chromophores, including melanin, hemoglobin, cytochromes, and nucleic acids [67,110,115]. Dyes like indocyanine green are another group of candidates [117]. All kinds of gold nanoparticles are the most popular molecular contrast agents for PT-OCT scanning due to their resonance in the NIR optical window. They are biocompatible, optical absorption tunable and can easily be used for photothermal therapy [104,106,107,112,113,115,116,172,173]. A discussion of different gold nanostructures can be found in Cho’s review [174]. Other demonstrated nanoparticle-based contrast agents include polypyrrole nanoparticles, carbon nanorods, and PLGA encapsulated dyes nanoparticles [67,175,176].

The merit of S-OCT is that it works along well with any molecule that owns a particular absorption spectrum characteristic in the NIR region, which means a vast range of choices of contrast agents candidates. Although endogenous contrast agents are mostly preferred, only a few endogenous chromophores are active in the NIR region. Theoretically, S-OCT can make use of any commercially available fluorescent dyes [123,131]. Besides, plasmon resonant nanoparticles with high extinction coefficients have been introduced to provide molecular information with S-OCT. Given the different sizes and geometry, the absorption peak of gold nanostructures can be tuned to match the OCT light source. Upconversion nanoparticles are also demonstrated as good molecular probes [114]. Overall, the contrast agents with solid absorption peaks in the NIR region are the most promising ones.

PS-OCT contrast agents should have at least one of the following polarization effects, namely preserved polarization, birefringence, diattenuation, and depolarization. Melanin and collagen are two reported endogenous contrast agents. Nanoparticles, especially gold nanoparticles, have a strong depolarization effect and therefore can be used as exogenous contrast agents.

In the ideal case, a good molecular contrast agent should have high selectivity for a biochemical target of interest, good stability and safety profile, signal enhancement, and time/cost efficiency [1]. However, most reported molecular OCT contrast agents have experienced limited success due to a lack of FDA approval. There is a constant concern about toxic properties during the history of contrast agent selection. Obviously, endogenous or FDA-approved contrast agents are able to prompt the corresponding molecular contrast OCT techniques to clinical applications. The nanomedicine development will accelerate the development of molecular contrast OCT approaches. Moreover, deep learning methods have been integrated to optimize the performance of nanoparticle-based contrast agents prior to clinical applications [177].

**Table 3:** Typical contrast agents for molecular contrast optical coherence tomography imaging in reported applications.

Name	Type	Modalities	Sample	Potential Applications	Citation
Melanin	Chromophore	PP-OCT	Scattering phantom	Melanoma	[42]
Melanin	Chromophore	PT-OCT	Pigmented mice eyes	Retinal disease	[115]
Melanocyte	Cell	S-OCT	*Xenopus laevis*	Nonspecific	[119]
Hemoglobin	Protein	PP-OCT	Multilayer phantom	Blood	[43]
Hemoglobin	Protein	S-OCT	In vivo mouse dorsal skin	Hypoxia and angiogenesis	[130]
Collagen	Protein	SH-OCT	Fish skin and young chicken wing bone	Cartilage and bone	[68]
Collagen	Protein	SH-OCT	Multilayer phantom	Skin	[70]
Collagen	Protein	SH-OCT	Rat-tail tendon	wound healing, aging, diabetes, and cancer	[71]
Collagen	Protein	SH-OCT	Fish scales, pig leg tendon, and rabbit eye sclera	In vivo endoscopic applications	[74]
Collagen	Protein	SH-OCT	Fish scales	Skin	[75]
Collagen	Protein	PS-OCT	Tendon and Zebrafish	Muscles	[142,143]
Lipid	Lipid	S-OCT	In vivo atherosclerotic rabbit model	Atherosclerosis	[128]
Blood plasma	Blood plasma	PT-OCT	Live rat ear	Vasculature system	[110]
Nerve fiber	Nerve fiber	PS-OCT	Nerve fibers in human eyes and rat model	Nerve system	[139,140]
dsRed	Protein	PP-OCT	Mouse mammary carcinoma cells	Breast cancer	[43]
Bacteriorhodopsin	Protein	Pump suppression OCT	Phantom	Nonspecific	[54]
Phytochrome A	Protein	Pump suppression OCT	Scattering phantom	Nonspecific	[55]
Benzene	Organic compound	NIVI	Phantom	Nonspecific	[59,61]
Acetone	Organic compound	NIVI	Phantom	Nonspecific	[62]
ICG	Dye	PP-OCT	Scattering phantom	Nonspecific	[41]
ICG	Dye	PT-OCT	Ex vivo pig eyes	Retinal surgery	[117]
ICG	Dye	S-OCT	*Xenopus laevis*	Nonspecific	[121]
MB	Dye	PP-OCT	Scattering phantom	Nonspecific	[45,54]
MB	Dye	PP-OCT	Live *Xenopus* tadpoles	Lymphatic and vasculature systems	[49]
ADS7460	Dye	S-OCT	Celery stalk	Cancer and Vasculature system	[120]
IR806	Dye	PT-OCT	Scattering phantom	Nonspecific	[108]
MB filled PLGA nanoparticles	Compound nanoparticle	PP-OCT	Human postmortem artery sections	Atherosclerosis	[51,52,53]
Gas vesicle	Compound nanoparticle	Engineered particle-based method	In vivo mouse retina	Nonspecific	[40]
Hematite (Fe_2_O_3_) microparticle	Magnetic particles	MM-OCT	Macrophages	Cancer	[88,89]
Magnetite microparticle	Magnetic particles	MM-OCT	In vivo *Xenopus laevis*	Nonspecific	[90,178]
Fe_3_O_4_ nanoparticle	Magnetic nanoparticle	MM-OCT	Zebrafish	Endoscopic imaging	[95]
Fe_3_O_4_ nanoparticle	Magnetic nanoparticle	MM-OCT	Mice breast tumor tissue	Cancer	[91]
Nano-screenMAG	Compound nanoparticles	MM-OCT	Scattering phantom and human mesenchymal stem cells	3D cell tracking in the retina	[179]
Polymer-coated magnetite nanoparticle	Compound nanoparticle	MM-OCT	Mice breast tumor tissue	Cancer	[91]
Polymer-coated Fe_3_O_4_ nanoparticle	Compound nanoparticle	MM-OCT	Live Swiss albino mice dorsal part skin	Nonspecific	[86]
Polymer-coated Fe_3_O_4_ nanoparticle	Compound nanoparticle	PT-OCT	Live Swiss albino mice dorsal part skin	Nonspecific	[86]
Dextran-coated Fe_3_O_4_ nanoparticle	Compound nanoparticle	MM-OCT	Melanoma mice model	Melanoma	[96]
Protein-coated iron oxide microparticle	Compound nanoparticles	MM-OCT	Scattering phantom	Nonspecific	[87]
Polyethylene glycol-covered iron oxides particle	Compound nanoparticle	MM-OCT	Ex vivo aorta specimens	Atherosclerosis	[94]
Iron-gold (Fe-Au) nanoparticle	Compound nanoparticle	MM-OCT	In vivo Swiss albino mice model	Melanoma	[100]
Superparamagnetic iron oxide nanoparticle	SPIONs	MM-OCT	Human cervical cancer (HeLa) cells	Cancer	[99]
amino-polyvinyl alcohol coated SPIONs	SPIONs	MM-OCT	Melanoma skin cancer samples of mice	Cancer	[97]
Magnetic graphene quantum dot nanoparticle	Compound nanoparticle	MM-OCT	3T3 cells	Tracking transplanted corneal stem cells	[98]
Iron oxide nanoparticle- encapsulated microsphere	Compound nanoparticle	MM-OCT	Cancer cells	Cancer	[101]
NaGdF_4_: Er^3+^/Yb^3+^ nanoparticle	Upconversion nanoparticles	PT-OCT	Chicken tissue	Pharmacokinetics and theranostics studies	[114]
Gold nanorod	Gold nanoparticle	S-OCT	Ex vivo human breast carcinoma	Breast cancer	[122]
Gold nanorod	Gold nanoparticle	PT-OCT	Live mouse ear	Cancer	[107]
Gold nanorod	Gold nanoparticle	PS-OCT	Lymphatics of the hind limb of mice	Cancer	[136]
Gold nanorod	Gold nanoparticle	PT-OCT	Sentinel lymph node of mice	Lymphatic system	[111]
Gold nanorod	Gold nanoparticle	PT-OCT	Pork tissue	Nonspecific	[112]
Gold nanorod	Gold nanoparticle	PT-OCT	Pigmented mice eyes	Retinal disease	[115]
ICAM2-targeted gold nanorod	Gold nanoparticle	PT-OCT	Live mice eyes	Sight threatening diseases	[116]
Gold nanosphere	Gold nanoparticle	PS-OCT	Phantom	Nonspecific	[163]
Gold nanosphere	Gold nanoparticle	PT-OCT	Live cells (MDA-MB-435 and MDA-MB-468)	Nonspecific	[104]
Gold nanoshell	Gold nanoparticle	Engineered particle-based method	HeLa and Jurkat cells	Cardiovascular disease	[38]
Gold nanoshell	Gold nanoparticle	PT-OCT	Phantom	Cancer	[103]
Gold nanoshell	Gold nanoparticle	PT-OCT	Ex vivo human breast tissue	Cancer markers	[106]
Gold nanocage	Gold nanoparticle	Engineered particle-based method	Mouse ear tumor	Cancer	[37]
Gold nanocage	Gold nanoparticle	S-OCT	Scattering phantom	Nonspecific	[124,125]
Antibody-conjugated gold nanoparticle	Compound nanopraticle	Engineered particle-based method	Excised hamster cheek pouch tissues	Cancer	[39]
Gold coated iron oxide nanorose	Compound nanoparticle	PT-OCT	Atherosclerotic lesions in rabbit arteries	Atherosclerosis	[105]
Gold nanobipyramid	Gold nanoparticle	PS-OCT	Live mouse	Nonspecific	[127]

## 12. Conclusions

In this paper, we reviewed current advances of molecular contrast OCT methods, summarized various contrast agents in corresponding applications, discussed the benefits and limitations of these techniques and contrast agents, and prospected the trends in this field. Although at its initial stage, molecular contrast OCT imaging may have a long-term clinical impact and a bright future in a wide range of nanomedical applications by adding sensitive molecular information to high-resolution morphological images.

## Figures and Tables

**Table 1:** Characteristics of some typical molecular imaging modalities.

Modality	Spatial Resolution	Temporal Resolution	Penetration Depth	Sensitivity	Multiplexing Capability	Safety Profile	Cost
**Positron emission tomography (PET)**	~5 mm	Seconds-minutes	>1 m	++	No	Ionizing radiation	+++
**Single photon emission tomography (SPECT)**	~10 mm	Minutes	>1 m	++	Yes	Ionizing radiation	+++
**Computed tomography (CT)**	~1 mm	Seconds-minutes	>1 m	+	No	Ionizing radiation	++
**Magnetic resonance imaging (MRI)**	~1 mm	Minutes-hours	>1 m	+	No	Good	+++
**Ultrasound (US)**	0.1–1 mm	Seconds-minutes	mm-cm	++	No	Good	+
**Bioluminescence imaging**	3–5 mm	Seconds-minutes	1–2 cm	+++	Yes	Good	+
**Fluorescence imaging**	0.5–3 mm	Seconds-minutes	<1 cm	++	Yes	Depends on fluorophore	+
**Photoacoustic imaging**	0.01–1 mm	Seconds-minutes	0.5–5 cm	++	Yes	Depends on imaging agents	+
**Surface-enhanced raman scattering imaging**	0.1–1 mm	Seconds-hours	5 mm	+++	Yes	Good	++
**Molecular contrast optical coherence tomography (MS-OCT)**	0.01 mm	Milliseconds-minutes	1 mm	+++	Depends on methods	Depends on extra fields and contrast agents	+

In this and succeeding tables, plus signs indicate relative amounts, i.e., + low, ++ medium, +++ high.

**Table 2:** Characteristics of the presented molecular contrast OCT techniques.

Technique	Signal Source	Spatial Resolution	Imaging Speed	Sensitivity	Extra Fields	Safety Profile	Multiplexing Capability	Contrast Agents
**Engineered particles**	Scattering and absorption	~10 µm	+++	+	None	Depends on contrast agents	Not yet	Exogenous
**PP-OCT**	Absorption	~10 µm	+	++	Pump light	Depends on contrast agents and pump light energy	Yes	Exogenous
**Pump suppression OCT**	Absorption	~15µm	+	+++	Pump light	Depends on contrast agents and pump light energy	Not yet	Exogenous
**SH-OCT**	Coherence	~15 µm	+	++	None	Depends on pump energy	Not yet	Endogenous
**NIVI**	Coherence	~15 µm	+	+++	Pump and Stokes light	Depends on pump energy	Yes	Exogenous and endogenous
**PT-OCT**	Phrase shift	~15 µm	+	++	Photothermal light	Depends on contrast agents and photothermal energy	Not yet	Exogenous and endogenous
**MM-OCT**	Phrase shift	~5 µm	+	++	Magnetic field	Depends on contrast agents	Not yet	Exogenous
**S-OCT**	Absorption	~35 µm	++	+++	None	Depends on contrast agents	Yes	Exogenous and endogenous
**PS-OCT**	Scattering	~10 µm	++	++	None	Depends on contrast agents	Not yet	Exogenous and endogenous

In this and succeeding tables, plus signs indicate relative amounts, i.e., + low, ++ medium, +++ high.
